# Temperature effect on the efficacy of 3 entomopathogenic nematode isolates against larvae of the lesser mealworm, *Alphitobius diaperinus* (Coleoptera: Tenebrionidae)

**DOI:** 10.1093/jee/toae292

**Published:** 2024-12-31

**Authors:** Eirini Karanastasi, Anna Nikorezou, Maria Stamouli, Anna Skourti, Maria C Boukouvala, Nickolas G Kavallieratos

**Affiliations:** Plant Protection Laboratory, Department of Agricultural Sciences, University of Patras, 30200 Messolonghi, Greece; Plant Protection Laboratory, Department of Agricultural Sciences, University of Patras, 30200 Messolonghi, Greece; Plant Protection Laboratory, Department of Agricultural Sciences, University of Patras, 30200 Messolonghi, Greece; Laboratory of Agricultural Zoology and Entomology, Department of Crop Science, Agricultural University of Athens, 11855 Athens, Greece; Laboratory of Agricultural Zoology and Entomology, Department of Crop Science, Agricultural University of Athens, 11855 Athens, Greece; Laboratory of Agricultural Zoology and Entomology, Department of Crop Science, Agricultural University of Athens, 11855 Athens, Greece

**Keywords:** darkling beetles, lesser mealworm, litter mealworm, entomopathogenic nematodes

## Abstract

The lesser mealworm *Alphitobius diaperinus* Panzer (Coleoptera: Tenebrionidae), an important insect pest of stored-product commodities and poultry production systems, is regarded among the most difficult species to control. It has developed resistance to many chemical insecticides, and though various cultural and biological methods have been assessed and identified as possible factors for its control, none are currently implemented. Entomopathogenic nematodes are often successfully employed as alternative to chemicals biocontrol agents of various insect species, including pests of stored products; nevertheless, their evaluation as potential biocontrol factors of the lesser mealworm is not efficiently scrutinized. In the current study, single *A. diaperinus* larvae were exposed to six doses of *Heterorhabditis bacteriophora* Poinar (Rhabditida: Heterorhabditidae), *Steinernema carpocapsae* (Weiser) (Rhabditida: Steinernematidae), and *Steinernema feltiae* (Filipjev) (Rhabditida: Steinernematidae), for 4 and 8 d, and mortality was recorded at 3 different temperature regimes, i.e., 25 ^o^C, 30 ^o^C, and 35 ^o^C. The study concludes that *S. carpocapsae* and *S. feltiae* are both highly virulent against *A. diaperinus* larvae and may be considered as promising biological control agents for reducing lesser mealworm infestations when applied at a rate of 70 IJs/cm^2^ at 25 ^o^C. When assessed at 30 ^o^C, both species were effective at the same rate though causing lower mortality of ~60% and ~50%, respectively, whereas their efficacy was low at 35 ^o^C.

## Introduction

Entomopathogenic nematodes (EPNs) are roundworm insect parasites that upon reaching the third infective stage of juveniles (IJ), they enter their host’s body through their spiracles, anus, and oral cavity, or occasionally through the cuticular intersegmental membrane, eventually killing the host (Kaya and Gaugler 1993, Singh et al. 2022). Nonetheless, they cannot be considered only as parasitic since, according to Griffin (2012), they can also enter the body of hosts killed by other causes, thus behaving as facultative parasites. Exploiting their parasitic properties, they can be integrated into a sustainable pest control strategy against stored product insect pests due to the absence of toxicity towards humans, nontarget organisms, and the environment (Ramos-Rodríguez et al. 2006, Athanassiou et al. 2010, Gulcu et al. 2017, Rumbos and Athanassiou 2017, Javed et al. 2020, Karanastasi et al. 2020). These nematodes are carriers of entomopathogenic bacteria, belonging primarily to the genera *Photorhabdus* and *Xenorhabdus*. Once the nematodes are inside the host, they regurgitate or defecate the bacteria through their mouth or anus, respectively. Subsequently, they proliferate within the nutrient-rich insect haemocoel. The bacteria secrete proteins and toxins, leading to septicemia and eventually death of the host insects (Boemare and Akhurst 2006). Additionally, recent reports suggest that *Steinernema* species can also contribute to insect mortality by releasing venom proteins (Chang et al. 2019).

EPN species belonging to the genera *Steinernema* of the family Steinermatidae and *Heterorhabditis* of the family Heterorhabditidae undergo a life cycle of 6 stages: egg, 4 juvenile stages (designated as J1–4), and adult. The third stage, also known as the infective juvenile (IJ), is pivotal as it marks the point at which the nematode enters the host’s body. Infective juveniles can be found in the soil, where they live freely and do not feed. Upon entering the host, IJs release their symbiotic bacteria that suppress the host’s immune system, leading to septicemic or toxemic death within 24–48 h. Insects succumbing to the *Steinernema–Xenorhabdus* complex typically exhibit a pale or grayish hue, varying with the EPN species, while those affected by the *Heterorhabditis–Photorhabdus* complex develop a dark-red coloration due to the bacteria’s production of luminescent anthraquinone pigments (Kaya and Gaugler 1993).

The most efficacious EPN species include *Steinernema carpocapsae* Weiser, *Steinernema feltiae* (Filipjev) (Rhabditida: Steinernematidae), and *Heterorhabditis bacteriophora* Poinar (Rhabditida: Heterorhabditidae) that are commercially available in several countries (Kavallieratos et al. 2022). These species have undergone extensive evaluations against adults and larvae of various stored-product insects. Numerous studies confirm their ability to suppress lepidopteran species, such as *Ephestia kuehniella* Zeller and *Plodia interpunctella* Hübner (Lepidoptera: Pyralidae), as well as coleopterans, such as *Oryzaephilus surinamensis* (L.) (Coleoptera: Silvanidae), *Tenebrio molitor* L., *Tribolium castaneum* Herbst (Coleoptera: Tenebrionidae), and *Trogoderma variabile* Ballion (Coleoptera: Dermestidae), in addition to adults of the lesser mealworm, *Alphitobius diaperinus* (Panzer) (Coleoptera: Tenebrionidae), the lesser grain borer, *Rhyzopertha dominica* (F.) (Coleoptera: Bostrychidae), and the rice weevil, *Sitophilus oryzae* (L.) (Coleoptera: Curculionidae) (Ramos-Rodríguez et al. 2006, Athanassiou et al. 2010, Zampara et al. 2014, Yuksel et al. 2019, Kavallieratos et al. 2022). Other EPN species, such as *Steinernema glaseri* (Steiner), *Steinernema riobrave* Cabanillas, Poinar, and Raulston, *Steinernema kraussei* Steiner, *Heterorhabditis indica* Poinar, Karunakar, and David, and *Heterorhabditis downesi* Stock, Griffin, and Burnell are also efficient and commercially available, however, not so commonly applied. From these, *S. glaseri* and *S. riobrave* have also been tested against stored-product insects (Alves et al. 2005, Rumbos and Athanassiou 2017), whereas some more studies have been conducted with *S. rarum* (Negrisoli et al. 2013, Del Valle et al. 2016), *S. bifurcatum* and *S. affine* (Khanum and Javed 2021), *H. indica*, *H. marelatus*, *H. megidis*, and *H. zealandica* (Mbata and Shapiro-Ilan 2005).

The lesser mealworm has recently been put in the spotlight, having been proposed as an alternative, sustainable nutrient source for food and feed applications (Rumbos et al. 2019, Rigopoulou et al. 2023). The ability of its larvae to efficiently convert a variety of agricultural byproducts and side-streams, into nutrient-rich biomass, as well as their rich in protein and other essential nutrients profile, makes them ideal ingredients for animal feed and human food. Moreover, their integration into sustainable food and feed production systems seems ideal (Varelas 2019, Siddiqui et al. 2024). With their high reproduction rate and minimal resource requirements, they present a compelling solution for enhancing food security while reducing environmental impact. However, the species may also pose a significant threat to agricultural and industrial settings, as it can infest stored grain commodities and is able to develop high populations in poultry houses and livestock facilities, causing damage to stored feed and creating unsanitary conditions (Sammarco et al. 2023). Additionally, it can transmit pathogens and parasites to animals, leading to reduced productivity and economic losses (Smith et al. 2022). Its high reproductive rate and ability to thrive in diverse environments make it particularly challenging to control. Effective management strategies, including sanitation, monitoring, and the use of insecticides, are essential to mitigate its impact on agricultural production systems (Sammarco et al. 2023). Recently, Kavallieratos et al. (2022) studied the efficacy of 3 EPN species against *A. diaperinus* adults among other stored product coleopterans and concluded that it was not affected by any of the EPN species used in the study. Their results confirm other studies that report very low mortality rates for *A. diaperinus* adults exposed to *S. carpocapsae* (Koc et al. 2022) and *H. bacteriophora* (Del Valle et al. 2016). In contrast, these works report that these EPN species may be used as control agents against *A. diaperinus* larvae, as also reported for *S. feltiae* by other studies (Fernandes et al. 2021). Nevertheless, the larvicidal effect of EPN against *A. diaperinus* has not been extensively studied, though they could be very successful and significant in managing this pest.

Considering the above, our study was conducted with the scope to provide information on the temperature-depending efficacy of 3 entomopathogenic nematode isolates against the lesser mealworm larvae.

## Materials and Methods

### Alphitobius diaperinus

The initial population of *A. diaperinus* derived from the Laboratory of Agricultural Zoology and Entomology (Agricultural University of Athens, Greece), where the species is maintained in the dark, at 30 ^o^C, 65% relative humidity (RH; Kavallieratos et al. 2022). To rear the insect, glass 5 L vials were half filled with wheat bran, yeast, and soy protein (6:1:1). One halved apple and a piece of polystyrene were added in each vial to provide a moisture source and supply protected pupation sites, respectively (Rice and Lambkin 2009). Before filling the vials, the bran was placed at −18 ^o^C for 48 h and subsequently at 50 ^o^C for 48 h to eradicate other possibly present arthropods.

Unsexed adult individuals were transferred to a 34 × 24 × 16 cm plastic container with one halved apple, filled with wheat flour, and left for 7 d at 26 ± 1 ^o^C, 55% RH for oviposition (Gourgouta et al. 2022). Then, the flour was sieved though a 250-μm sieve and the eggs were transferred into a glass vial filled with rearing mixture. The vials were dated so that each one contained coeval larvae which were subsequently used for the bioassays.

## Entomopathogenic Nematode Isolates

The EPNs utilized in the current study, i.e., *H. bacteriophora*, *S. carpocapsae*, and *S. feltiae*, were originally provided by Bio-insecta (Nea Silata, Greece). For the bioassays, the nematodes were multiplied in vivo, at 25 °C in larvae of the greater wax moth, *Galleria mellonella* (L.) (Lepidoptera: Pyralidae) as described by Kaya and Stock (1997) and stored at ~10 °C in cell culture flasks until use. Prior to each bioassay, the vitality of the nematodes was checked under a stereomicroscope; however, none was used after having been stored for more than 4 wk.

## Bioassays

The EPN doses that were used in the current study were 100, 500, 1,000, 5,000, 10,000, and 50,000 IJs/mL, following the custom experimentation protocol adopted by our research team (Karanastasi et al. 2020, Kavallieratos et al. 2022). The experiments were conducted with single *A. diaperinus* larvae, each transferred in a 300-mm plastic petri dish, lined with filter paper. For each dose and each EPN species, the bioassay comprised 3 replications of 10 petri dishes, each containing 1 unsexed small larva (<7 mm), at 65% RH.

Each petri dish was inoculated with a given EPN dose and applied with 100 μL of tap water containing 10, 50, 100, 500, 1,000, and 5,000 IJs/larva, equivalent to the aforementioned doses. The EPN doses were freshly prepared for each replication. Control petri dishes were inoculated with 100-μL tap water.

Larval mortality was recorded after 4 and 8 d of exposure. A larva was declared diseased when after poking with a brush, no motion was detected. Dead individuals were removed from the petri dishes and transferred to white traps, separately for each bioassay. EPN infection was confirmed with the emergence of EPN IJs within the following 2 wk. Additionally, in case of larvae that had turned into pupae, after 8-d exposure, these were singly maintained for observation at the respective temperature regime. All these pupae developed to adults.

The whole experiment was repeated 3 times for each temperature regime, specifically at 25 ^o^C, 30 ^o^C, and 35 ^o^C. In total, 5130 individuals were used in the experiments, i.e., [(30 larvae × 3 EPN species × 6 EPN doses) plus controls] × 3 repetitions × 3 temperatures.

## Statistical Analysis

Mortality was adjusted following Abbott’s method (Abbott 1925). Initially, all data were checked for normality and homogeneity of variances utilizing Shapiro–Wilk and Levene’s tests, respectively. Since the same vials were checked for mortality after 4 and 8 d of exposure, the mortality data were analyzed using a repeated measures ANOVA, with exposure as the repeated measure variable and nematode formulation, dose rate, and temperature as the main effects, using the JPM 7 software (SAS Institute Inc., Cary, NC, USA). For each nematode isolate, exposure interval and within each dose rate, the mortality data were subjected to ANOVA to identify differences among temperatures. Additionally, for each exposure interval, nematode formulation and within each temperature, the mortality data were analyzed with ANOVA to determine differences among dose rates. Means were separated using the Tukey HSD test at the 5% significance level.

## Results

In general, the control treatment mortality was low and did not exceed 20% in any of the cases. Mortality of *A. diaperinus* larvae was significantly affected by the exposure interval (*F* = 45.6, df = 1, *P* < 0.001) and all main effects, i.e., nematode isolate (*F* = 16.3, df = 2, *P* < 0.001), dose rate (*F* = 13.4, df = 5, *P* = 0.004), as well as temperature (*F* = 18.5, df = 2, *P* < 0.001), whereas with few exceptions, all associated interactions were not significant (Table 1).

**Table 1.** Repeated measures ANOVA parameters for main effects and associated interactions for mortality levels of *Alphitobius diaperinus* exposed for 4 and 8 d on 3 commercial formulations of entomopathogenic nematodes (*S. carpocapsae*, *S. feltiae*, and *H. bacteriophora*) applied at six dose rates (10, 50, 100, 500, 1,000, and 5,000 IJs/larva) at 3 temperatures (25, 30, and 25°C) (error df: 108)

Source	df	*F*	*P*
*Source between variables*			
All between	53	5.1	<0.001
Intercept	1	552.3	<0.001
Nematode formulation	2	16.3	<0.001
Dose rate	5	13.4	0.004
Temperature	2	18.5	<0.001
Nematode formulation × Dose rate	10	0.7	0.737
Nematode formulation × Temperature	4	8.4	<0.001
Dose rate × Temperature	10	7.4	<0.001
Nematode formulation × Dose rate × Temperature	20	0.9	0.547
*Source within variables*			
Within interactions	53	1.5	0.051
Exposure	1	45.6	<0.001
Exposure × Nematode formulation	2	0.3	0.720
Exposure × Dose rate	5	0.4	0.858
Exposure × Temperature	2	31.6	<0.001
Exposure × Nematode formulation × Dose rate	10	0.3	0.984
Exposure × Nematode formulation × Temperature	4	0.5	0.755
Exposure × Dose rate × Temperature	10	0.3	0.978
Exposure × Nematode formulation × Dose rate × Temperature	20	0.2	1.000

### Steinernema feltiae

After 4 d of *A. diaperinus* larvae exposure to *S. feltiae* at 25 ^o^C, low mortality (<25%) was observed when 10 and 50 IJs/larva were applied (Table 2), though it increased to 77.4% when 100 IJs/larva were applied. A higher mortality of up to 96.4% was achieved with the higher dose rates (500, 1,000, and 5,000 IJs/larva); however, this effect was not significant. After 8 d of exposure at the same temperature, mortality followed the same pattern and ranged between 79.5% and 96.2% for dose rates higher than 100 IJs/larva.

**Table 2.** Mean mortality ± SE of *Alphitobius diaperinus* larvae exposed for 4 and 8 d on a commercial formulation of the entomopathogenic nematode *S. feltiae* applied at six dose rates (10, 50, 100, 500, 1,000, and 5,000 IJs/larva) at 3 temperatures (25, 30, and 25°C).

Temperature	Dose rate (IJs/larva)	Mortality (%)	
		Days post application	
		4	8
** 25°C**	10	1.2 ± 1.2 B	2.6 ± 1.3 B
	50	25.0 ± 14.4 B	24.4 ± 17.2 B
	100	77.4 ± 4.3 Aa	79.5 ± 6.4 A
	500	94.1 ± 3.1 Aa	93.6 ± 3.4 A
	1,000	96.4 ± 3.6 Aa	96.2 ± 3.8 A
	5,000	82.1 ± 9.4 Aa	80.8 ± 10.2 A
** 30°C**	10	14.3 ± 9.5	16.0 ± 8.3
	50	6.0 ± 6.0	15.2 ± 8.3
	100	22.6 ± 17.5 b	26.2 ± 18.6
	500	41.7 ± 18.5 b	44.3 ± 22.2
	1,000	50.0 ± 10.9 b	59.5 ± 18.4
	5,000	59.5 ± 20.7 ab	58.2 ± 21.1
** 35°C**	10	31.0 ± 22.2	45.6 ± 22.4
	50	6.0 ± 3.1	37.6 ± 21.21
	100	3.6 ± 0.0 b	35.4 ± 20.1
	500	11.9 ± 6.3 b	28.7 ± 19.5
	1,000	11.9 ± 8.6 c	35.4 ± 19.0
	5,000	9.5 ± 2.4 b	34.2 ± 21.5

For each exposure interval, within each temperature, means followed by the same uppercase letter do not differ significantly (in all cases df = 5, 17, Tukey HSD test at *P* = 0.05). For each exposure interval, within each dose rate, means followed by the same lower-case letter are not significantly different (in all cases, df = 2, 8, Tukey HSD test at *P *= 0.05). Where no letters exist, no significant differences were noted.

At 30 ^o^C, mortality after 4 d of exposure was significantly reduced compared with 25 ^o^C. Indicatively, the highest mortality hardly reached 59.5% when 5000 IJs/larva were applied. After 8 d of exposure, the highest mortality was recorded after the application of 1,000 (59.5%) and 5,000 IJs/larva (58.2%); however, differences observed among different dose rates and temperatures were not significant.

The lowest mortality levels of *A. diaperinus* larvae achieved by *S. feltiae* were recorded at 35 ^o^C (Table 2). Specifically, after 4 d of exposure mortality did not exceed 31.0%, and no significant differences were noted among different dose rates. Similar effects were observed 8 d after application.

### Steinernema carpocapsae

When *A. diaperinus* larvae were exposed to *S. carpocapsae* at 25 ^o^C, the highest mortality after 4 d was recorded for the dose rates 500, 1,000, and 5,000 IJs/larva (82.1%, 85.7%, and 92.9%, respectively), being significantly different than the respective mortalities noted for the lower dose rates (Table 3). In all cases, mortality was not further increased after 8 d of exposure, following the same pattern among dose treatments.

**Table 3.** Mean mortality ± SE of *Alphitobius diaperinus* larvae exposed for 4 and 8 d on a commercial formulation of the entomopathogenic nematodes *S. carpocapsae* applied at six dose rates (10, 50, 100, 500, 1,000, and 5,000 IJs/larva) at 3 temperatures (25, 30, and 25°C)

Temperature	Dose rate (IJs/larva)	Mortality (%)	
		Days post application	
		4	8
** 25°C**	10	10.7 ± 7.4 C	10.3 ± 6.8 C
	50	36.9 ± 6.0 BC	38.5 ± 4.4 B
	100	40.5 ± 2.4 B	41.0 ± 3.4 B
	500	82.1 ± 6.2 Aa	82.1 ± 5.6 A
	1,000	85.7 ± 7.4 Aa	85.9 ± 7.1 Aa
	5,000	92.9 ± 3.6 Aa	92.3 ± 3.8 Aa
** 30°C**	10	17.9 ± 10.3	22.8 ± 12.5
	50	48.8 ± 11.4	54.4 ± 17.5
	100	48.8 ± 17.2	48.1 ± 20.7
	500	63.1 ± 10.4 a	69.6 ± 15.3
	1,000	75.0 ± 18.0 a	74.7 ± 19.9 ab
	5,000	81.0 ± 17.3 a	79.7 ± 18.4 ab
** 35°C**	10	28.6 ± 18.3	43.0 ± 19.5
	50	15.5 ± 12.1	39.2 ± 19.1
	100	21.4 ± 12.5	45.6 ± 12.7
	500	17.9 ± 7.1 b	38.0 ± 12.7
	1,000	14.3 ± 7.5 b	25.3 ± 8.3 b
	5,000	4.8 ± 4.8 b	21.1 ± 20.5 b

For each exposure interval, within each temperature, means followed by the same uppercase letter do not differ significantly (in all cases df = 5, 17, Tukey HSD test at *P* = 0.05). For each exposure interval, within each formulation and dose, means followed by the same lower-case letter are not significantly different (in all cases, df = 2, 8, Tukey HSD test at *P* = 0.05). Where no letters exist, no significant differences were noted.

At 30 ^o^C, mortality caused by *S. carpocapsae* after 4 d of exposure, no statistically significant differences among dose rates were observed. For the dose rates 500, 1,000, and 5,000 IJs/larva, mortality was slightly lower as compared with 25 ^o^C; however, this effect was not significant. A similar trend was observed for the mortality rates recorded after 8 d.

Similar to *S. feltiae*, mortality caused by *S. carpocapsae* at 35 ^o^C was generally low and did not exceed 28.6% and 45.6% after 4 and 8 d of exposure, respectively. Mortality levels after the application of 1,000 and 5,000 IJs/larva were significantly different from the respective figures at 25 ^o^C and 30 ^o^C for both exposure intervals (4 and 8 d). No statistically significant differences though were observed among dose rates.

### Heterorhabditis bacteriophora

The application of 10 and 50 IJs/larva *H. bacteriophora* at 25 ^o^C resulted in low mortality for both exposure intervals (<6%) (Table 4). The highest mortality after 4 d of exposure at 25 ^o^C was observed at the application of 500 IJs/larva (61.9%), although not statistically different from 1,000 (31.0) and 5,000 IJs/larva (56.0%). After 8 d of exposure, mortality was generally further increased, reaching 76.9% for the 500 IJs/larva dose. At the higher temperatures, mortality was generally low, hardly reaching 34.5 (4 d) and 34.2% (8 d) at 30 ^o^C, and 32.1 (4 d) and 51.9% (8 d) at 35 ^o^C, respectively.

**Table 4.** Mean mortality ± SE of *Alphitobius diaperinus* larvae exposed for 4 and 8 d on a commercial formulation of the entomopathogenic nematodes *H. bacteriophora* applied at six dose rates (10, 50, 100, 500, 1,000, and 5,000 IJs/larva) at 3 temperatures (25, 30, and 25°C)

Temperature	Dose rate (IJs/larva)	Mortality (%)	
		Days post application	
		4	8
** 25°C**	10	0.0 ± 0.0 Bb	1.3 ± 1.3 C
	50	6.0 ± 3.1 B	5.1 ± 2.6 Cb
	100	22.6 ± 6.3 AB	20.5 ± 4.6 BC
	500	61.9 ± 13.1 Aa	76.9 ± 2.2 Aa
	1,000	31.0 ± 15.7 AB	29.5 ± 18.5 BC
	5,000	56.0 ± 7.2 Aa	56.4 ± 7.8 AB
** 30°C**	10	3.6 ± 3.6 Bab	5.5 ± 5.5 B
	50	11.9 ± 1.2 AB	11.4 ± 3.4 ABab
	100	8.3 ± 5.2 AB	5.9 ± 3.7 B
	500	13.1 ± 7.2 ABb	11.0 ± 5.9 ABb
	1,000	19.0 ± 9.3 AB	17.7 ± 7.7 AB
	5,000	34.5 ± 7.2 Aab	34.2 ± 5.5 A
** 35°C**	10	15.5 ± 4.3 a	35.4 ± 13.2
	50	19.0 ± 9.5	48.1 ± 15.6 a
	100	32.1 ± 20.9	51.9 ± 21.6
	500	10.7 ± 5.5 b	36.7 ± 22.1 ab
	1,000	21.4 ± 12.5	48.1 ± 14.6
	5,000	15.5 ± 10.2 b	32.9 ± 20.4

For each exposure interval, within each temperature, means followed by the same uppercase letter do not differ significantly (in all cases df = 5, 17, Tukey HSD test at *P* = 0.05). For each exposure interval, within each formulation and dose, means followed by the same lower-case letter are not significantly different (in all cases, df = 2, 8, Tukey HSD test at *P* = 0.05). Where no letters exist, no significant differences were noted.

## Discussion

Entomopathogenic nematodes (EPNs) are widely known as effective biological control agents (BCAs) against many insects, being excellent substitutes for chemical control; however, their success depends on multiple parameters. One such parameter is insect behavior, pertaining to the tactics of the nematode species, i.e., ‘cruisers’ move actively through the soil in search of their host, ‘ambushers’ lie in waiting for the insect to approach, and ‘intermediates’ combine the 2 tactics. Another factor is insect’s susceptibility to the nematode symbiotic bacterial species or strain that actually induces host death. Additionally, EPNs are susceptible and dependent on prevailing conditions, such as humidity and temperature, especially in soil-less environments. Therefore, their effectiveness as BCAs must be thoroughly investigated at differential conditions for each possible target. For example, *S. carpocapsae* is an unspecific parasite capable of infecting more than 250 insect species from at least 13 orders (Poinar 1979, Hodson et al. 2011). Lu et al. (2017), who studied the species secretome, found that it releases more than 450 different proteins when initiating active parasitism, many of which have been hypothesized as involved in host tissue damage and immunosuppression. Another generalist, *S. feltiae*, has a more limited host range (Spiridonov et al. 2004, Adams et al. 2007), whereas others, such as *S. scapterisci* and *S. scarabaei*, infect a much narrower insect range (Nguyen and Smart 1990, Stock and Koppenhöfer 2003).

With respect to EPN control potential against the lesser mealworm *Alphitobius diaperinus*, only a few studies have been conducted (Geden and Axtell 1988, Alves et al. 2005, Del Valle et al. 2016, Fernandes et al. 2021, Kavallieratos et al. 2022, Koc et al. 2022). This species is a significant pest of stored grain products and feed, as well as of livestock and poultry facilities, causing substantial damage and creating unsanitary conditions (Sammarco et al. 2023). Those former studies show ambiguous and sometimes contradicting results, though it is quite evident that *A. diaperinus* adults are poorly or less sensitive to EPN infection comparing to their larvae (Del Valle et al. 2016, Kavallieratos et al. 2022, Koc et al. 2022). This may be due to several factors such as exoskeleton structure (Silva Soares et al. 2018), high motility, spiracle aperture size, or others. Hence, the current study intended to shed more light on EPN efficacy against *A. diaperinus* larvae and investigate the impact of prevailing temperature.

The results of the present study show that mortality rates of *A. diaperinus* larvae were very low at the lowest dose rates (10 and 50 IJs/larva), while they increased at dose rates equal or higher than 100 IJs/larva. Nonetheless, in most instances, mortality at 100 IJs/larva was not significantly different to that caused by higher dose rates, except for *S. carpocapsae* and *H. bacteriophora* at 25 ^o^C after 4 d of exposure, which was higher at 500 IJs/larva. These findings are in accordance with other published works, as, for example, with Fernandes et al. (2021) who screened 16 EPN isolates from which, the highest (98%) mortality was observed with *S. feltiae* at concentrations of 30 and 50 JIs/cm² (i.e., ~114–190 IJs/insect) at 25 ^o^C. Of course, as described in former studies, the efficacy of EPNs against *A. diaperinus* depends on the nematode isolate (Alves et al. 2012), which in fact means that it is affected by the symbiont’s bacterial strain, and *vice versa*, suggesting that different insect strains have variable susceptibility to the same EPN isolate (Kavallieratos et al. 2022). It is also possible that native EPN isolates have different performance against insects of the same geographic origin (Fernandes et al. 2021). In the present study, the 2 *Steinernema* isolates resulted to a mortality rate higher than 90% at 25 ^o^C, whereas *H. bacteriophora* was less effective against the lesser mealworm.

Importantly, to date, the scarce work that has been published with respect to EPN control efficacy against *A. diaperinus* has been conducted at 25−27 ^o^C, which is a usual and more importantly, recommended temperature range for EPN applications. However, the lesser mealworm is naturally thriving on the floor of poultry houses, in litter, grains, milled products, or spoiling food, where the temperature is often higher. Moreover, *A. diaperinus* larvae perform better at elevated temperatures, e.g., 30−32 °C (Kotsou et al. 2021). During the present study, we tested the divergence of EPN effectiveness at 3 different temperature regimes and we unequivocally demonstrated that all 3 EPN species are more effective at 25 ^o^C against *A. diaperinus* larvae as mortality rates decreased at the higher temperatures (30 ^o^C and 35 ^o^C) employed. This result is in accordance with Geden and Axtell (1988), who reported a rapid decline of *S. feltiae* numbers at temperatures higher than 24 ^o^C after 5 wk. Additionally, mortality did not increase after 8 d of exposure as compared with 4 d of exposure, which is in accordance with the common behavior and effect of EPNs that kill their hosts within 48 h after entering its body.

To summarize the above, we conclude that *S. carpocapsae* and *S. feltiae* are both highly virulent against *A. diaperinus* larvae, causing high larval mortality, and may be used as promising BCAs for reducing lesser mealworm infestations when applied at a rate of 500 IJs/larva, i.e., 70 IJs/cm^2^ at 25 ^o^C. When higher temperatures (~30 ^o^C) were tested, both species were effective at this dose rate and, however, presented lower mortality of ~60% and ~50%, respectively. Importantly, the competence of EPNs to kill *A. diaperinus* larvae, considering also that they multiply in the larval cadaver and hence can infect more individuals, may provide us with an efficient biocontrol tool for reducing the pest’s population. For pests such as the lesser mealworm, whose adults have a 3*–*5 fold life span comparing to their larvae, while giving birth to a total of 200–400 eggs (Sammarco et al. 2023), it is crucial to eradicate it before giving birth to the next generation. Additionally, since EPNs infect only insects or arthropods connected with insects, their use does not pose any threat to animals or humans, and thus, safety concerns for food facilities and livestock are not raised.

Contrarywise, *H. bacteriophora* was not successful in eradicating the lesser mealworm, neither at the larval stage as shown in the present study nor against adults as shown in our previous work (Kavallieratos et al. 2022); consequently, it is not recommended for its control.
